# Laparoscopic gastrectomy in obese gastric cancer patients: a comparative study with non-obese patients and evaluation of difference in laparoscopic methods

**DOI:** 10.1186/s12876-017-0638-1

**Published:** 2017-06-19

**Authors:** Ke Chen, Yu Pan, Shu-ting Zhai, Jia-qin Cai, Qi-long Chen, Ding-wei Chen, Yi-ping Zhu, Yu Zhang, Ya-ping Zhang, Hendi Maher, Xian-fa Wang

**Affiliations:** 1 Department of General Surgery, Sir Run Run Shaw Hospital, School of Medicine, Zhejiang University, 3 East Qingchun Road, Hangzhou, Zhejiang Province 310016 China; 2 Department of Gastroenterology, Sir Run Run Shaw Hospital, School of Medicine, Zhejiang University, 3 East Qingchun Road, Hangzhou, Zhejiang Province 310016 China; 3 Department of Anesthesiology, Sir Run Run Shaw Hospital, School of Medicine, Zhejiang University, 3 East Qingchun Road, Hangzhou, Zhejiang Province 310016 China; 4 School of Medicine, Zhejiang University, 866 Yuhangtang Road, Hangzhou, Zhejiang Province 310058 China

**Keywords:** Laparoscopic gastrectomy, Stomach neoplasms, Obesity, Body mass index, Morbidity

## Abstract

**Background:**

Obesity is a growing epidemic around the world, and obese patients are generally regarded as high risk for surgery compared with normal weight patients. The purpose of this study was to evaluate the influence of obesity on the surgical outcomes of laparoscopic gastrectomy (LG) for gastric cancer.

**Methods:**

We reviewed data for all patients undergoing LG for gastric cancer at our institute between October 2004 and December 2016. Patients were divided into non-obese and obese groups and the perioperative outcomes were compared. Furthermore, a subgroup analysis was conducted to evaluate which of the two commonly used methods of LG, laparoscopic-assisted gastrectomy (LAG) and totally laparoscopic gastrectomy (TLG), is more suitable for obese patients.

**Results:**

A total of 1691 patients, 1255 non-obese and 436 obese or overweight patients, underwent LG during the study period. The mean operation time was significantly longer in the obese group than in the non-obese group (209.9 ± 29.7 vs. 227.2 ± 25.7 min, *P* < 0.01), and intraoperative blood loss was significantly lower in the non-obese group (113.4 ± 34.1 vs. 136.9 ± 36.7 ml, *P* < 0.01). Time to first flatus, time to oral intake, and postoperative hospital stay were significantly shorter in the non-obese group than in the obese group (3.3 ± 0.8 vs. 3.6 ± 0.9 days; 4.3 ± 1.0 vs. 4.6 ± 1.0 days; and 9.0 ± 2.2 vs. 9.6 ± 2.2 days, respectively; *P* < 0.01). 119 (9.5%) of the non-obese patients had postoperative complications as compared to 44 (10.1%) of the obese patients (*P* = 0.71). In the subgroup analysis of all patients, TLG showed improved results for early surgical outcomes compared to LAG, mainly due to its advantages in obese patients.

**Conclusions:**

Obesity is associated with long operation time, increased blood loss, and slow recovery after laparoscopic gastric resection but does not affect intraoperative security or effectiveness. TLG may have less negative results in obese patients than LAG due to a variety of reasons. Our analysis shows that TLG is more advantageous, with regard to early surgical outcomes, for obese patients.

## Background

Gastric cancer, as the third major course of the world’s cancer-related death, is still the fifth most commonly seen cancer, despite the decreased mortality [1]. Radical gastrectomy with regional lymph node dissection is the only potentially available curative therapy though the survival of the patients is improved by adjuvant chemotherapy [2–5]. Since laparoscopic gastrectomy (LG) was first reported in 1994 for the treatment of early stage gastric adenocarcinoma [6], this technique has been rapidly adopted within East Asia. The advantages of LG include decreased pain, better cosmesis, faster recovery, fewer complications, and quicker return to normal movement compared to open surgery [7–15]. Furthermore, evidence continues to show no difference between patients undergoing open or laparoscopic surgery for oncologic outcomes [16–18]. The two commonly used approaches of LG for gastric cancer are totally laparoscopic gastrectomy (TLG) and laparoscopic-assisted gastrectomy (LAG). Perigastric lymphadenectomy in both techniques can be conducted under the laparoscopy. Nevertheless, an intracorporeal anastomosis characterizes the former one without the need of an auxiliary incision while the latter one needs an epigastrium auxiliary incision for safe en bloc extraction of the specimen to complete reconstruction of the digestive tract.

As one of the most essential health issues in the world, obesity has been widely known to aggravate numerous medical issues [19], for instance, hypertension, diabetes mellitus, lipid disorder, and obstructive sleep apnea, all of which can affect the surgical outcomes negatively [20]. In fact, the rate of obesity continues to increase worldwide in spite of the negative effects on human health. Therefore, laparoscopic gastric surgeons tend to see a growing number of overweight or obese patients, which has, and will continue to, increase complications. [21–26]. Thus, the advantages which have been mentioned previously have enhanced the application of the LG to high-risk patients, like obese people, who suffer from increased morbidity after open gastrectomy in comparison with normal weight patients. [23–26]. With respect to the laparoscopic method, some have found increased complication rates or slower recovery [27, 28], whereas some have reported equivalent benefits and postoperative outcomes following LG in obese patients in comparison with non-obese ones [29–33]. Thus, it remains uncertain whether the use of a laparoscopic method can reduce the difference in the morbidity or recovery between non-obese and obese patients who are suffering from gastric cancer and receiving gastrectomy. Besides, it is reported that TLG could contribute to the improvement of early surgical outcomes in overweight or obese patients compared to LAG [34]. However, such results are still not confirmed by large sample studies. Therefore, this study aimed to evaluate the effect of obesity on outcomes in laparoscopic gastric surgery based on results from our high volume center. Concurrently, subgroup analysis was carried out to determine which was more suitable of the two LG methods for obese gastric cancer patients.

## Methods

### Patients

A retrospective review of patients receiving LG from a prospectively maintained database of gastric adenocarcinoma disease between October 2004 and December 2016 at Sir Run Run Shaw Hospital was performed. All surgeons in our institution have been performing laparoscopic surgery for over 2 years, and the seniors had more than 5 years of experience. Written consent was acquired from everyone before enrollment in the study. This research was approved by the Zhejiang University’s Ethics Committee.

### Surgical procedure

With the patient in the supine position, mobilization of the stomach and en bloc systematic lymph node dissection was performed via five trocars under a pneumoperitoneum. Total or distal gastrectomy was performed, according to tumor location, size, and depth of invasion. Proximal gastrectomy was not used in our center because of the relatively higher rate of reflux esophagitis. D_2_ lymphadenectomy was undertaken complying with the rules of the Gastric Cancer Treatment Guidelines 2011 by the Japanese Gastric Cancer Association [35]. Total gastrectomy was performed with spleen preservation. Anastomosis was completed extracorporeally or intracorporeally. Initially, an epigastrium auxiliary incision was made to facilitate the excision of the specimen and the reconstruction of the digestive tract. However, subsequent advancement in laparoscopic instruments and increased experience in the performance of intricate laparoscopic gastrointestinal procedures made us start using intracorporeal anastomosis by stapler or hand-sewn techniques. The surgical procedures are described in detail in our previously published articles [36–39].

### Patient data evaluation

According to preoperative body mass index (BMI), patients were assigned to the obese group (BMI >25 kg/m^2^) and the non-obese (BMI 25 < = kg/m^2^). Despite the World Health Organization’s definition of obesity being a BMI over 30 kg/m^2^, we used 25 kg/m^2^ as our cutoff because the average BMI for Asian people is lower than the BMI for non-Asian people, especially when compared to Western populations. Statistics about the demographics of patients, postoperative outcomes and the surgical procedure were gathered. Clinical and pathological staging were determined according to the tumor-node-metastasis (TNM) model and the American Joint Committee on Cancer (the 7th edition). Comparisons were conducted between the two groups, regarding estimated blood loss (EBL), length of hospital stay, duration of operation, time to oral intake, time to flatus, amount of retrieved lymph nodes, mortality and morbidity.

### Subgroup analysis

To evaluate the benefits of the two different LG approaches, relevant differences between LAG and TLG cases were compared among obese and non-obese patients. Furthermore, LAG and TLG cases were divided into laparoscopic-assisted distal gastrectomy (LADG), laparoscopic-assisted total gastrectomy (LATG) and totally laparoscopic distal gastrectomy (TLDG), totally laparoscopic total gastrectomy (TLTG), respectively, which were also compared between obese versus non-obese patients to confirm whether or not the possible benefits are related to surgical extension.

### Statistical analysis

Results were presented as mean ± standard deviations (SDs). Continuous variables were compared using the Student’s t test and categorical variables were compared with χ^2^ test or the Fisher exact probability test. Differences with *P* values (*P* < 0.05) were considered statistically significant. All statistical analyses were performed with SPSS software, version 18.0 (SPSS Inc., Chicago, United States).

## Results

### Patient characteristics and pathological features

Table 1 shows the clinical characteristics and pathologic features of the patients. A total of 1691 patients, 1255 non-obese and 436 obese patients, underwent LG during the study period. The proportion of males was higher in the obese cohort than in the non-obese cohort (61.9% vs. 72.4%, *P* < 0.01). Otherwise, the two cohorts were similar with respect to preoperative risk factors such as age, the American Society of Anesthesiologists’ score (ASA), tumor size and TNM stage. However, more patients in the non-obese cohort underwent total gastrectomy (30.8% vs. 22.9%, *P* < 0.01).

**Table 1:** Comparison of the clinicopathological characteristics

Variable	Non-obese (*n* = 1255)	Obese (*n* = 436)	*P* value
Age (years)	58.2 ± 11.1	58.0 ± 11.0	0.78
Gender (M/F)	777/478	320/116	<0.01
ASA classification (I/II/III)	593/610/52	226/182/28	0.02
Tumor size (cm)	3.7 ± 1.7	3.7 ± 1.7	0.91
TNM stage (I/II/III)	655/275/325	211/103/122	0.39
Surgical extension (D/T)	869/386	336/100	<0.01

*M* male, *F* female, *D* distal gastrectomy, *T* total gatrectomy

### Intraoperative and postoperative outcomes

The intraoperative findings and subsequent postoperative recovery are displayed in Table 2. The mean operation time in the obese cohort was 17.3 min longer than for non-obese (209.9 ± 29.7 vs. 227.2 ± 25.7 min, *P* < 0.01). Intraoperative blood loss was lower in the non-obese cohort than the obese one (113.4 ± 34.1 vs. 136.9 ± 36.7 ml, *P* < 0.01). The difference in the number of harvested lymph nodes between groups was not significant (35.3 ± 9.8 vs. 34.7 ± 8.7, *P* = 0.27). The mean time to first flatus was shorter in the non-obese cohort than in the obese cohort (3.3 ± 0.8 vs. 3.6 ± 0.9 days, *P* < 0.01), similarly the early time to restart diet postoperatively (4.3 ± 1.0 vs. 4.6 ± 1.0 days, *P* < 0.01). A shorter length of hospital stay was also observed in the non-obese cohort (9.0 ± 2.2 vs. 9.6 ± 2.2 days, *P* < 0.01).

**Table 2:** Comparison of surgical outcomes and postoperative recovery

Variable	Non-obese (*n* = 1255)	Obese (*n* = 436)	*P* value
Operation time (min)	209.9 ± 29.7	227.2 ± 25.7	<0.01
Blood loss (mL)	113.4 ± 34.1	136.9 ± 36.7	<0.01
Number of retrieved lymph nodes	35.3 ± 9.8	34.7 ± 8.7	0.27
Time to first flatus (days)	3.3 ± 0.8	3.6 ± 0.9	<0.01
Time to starting liquid diet (days)	4.3 ± 1.0	4.6 ± 1.0	<0.01
Postoperative hospital stay (days)	9.0 ± 2.2	9.6 ± 2.2	<0.01

There was no postoperative mortality in both non-obese cohort and obese cohort. The complications after surgery are listed in Table 3. The morbidity rate in the non-obese cohort was 9.5% (119/1255 patients) and 10.1% (44/436 patients) in the obese cohort, overall difference was not significant (*P* = 0.71). The leading complications in the non-obese cohort were abdominal abscess (17 cases, 1.4%) and stasis (18 cases, 1.4%). Other complications were anastomotic leakage (*n* = 5), anastomotic stricture (*n* = 5), anastomotic bleeding (*n* = 8), intracorporeal hemorrhage (*n* = 11), pancreatic leakage (*n* = 14), ileus (*n* = 4), lymphorrhea (*n* = 14), and wound infection (*n* = 2). The most common complications in the obese cohort were also abdominal abscess (7 cases, 1.6%) and stasis (11 cases, 2.5%). In addition, ileus (6 cases, 1.4%) and wound infection (5 cases, 1.1%) were relatively common in obese patients. Other complications included anastomotic leakage (*n* = 1), anastomotic bleeding (*n* = 1), intracorporeal hemorrhage (*n* = 2), and lymphorrhea (*n* = 3).

**Table 3:** Comparison of postoperative complications

Variable	Non-obese (*n* = 1255)	Obese (*n* = 436)	*P* value
Overall complications (%)	119 (9.5)	44 (10.1)	0.71
Anastomotic leakage (%)	5 (0.4)	1 (0.2)	
Anastomotic stricture (%)	6 (0.5)	0 (0.0)	
Anastomotic bleeding (%)	8 (0.6)	1 (0.2)	
Intracorporeal hemorrhage (%)	11 (0.9)	2 (0.5)	
Abdominal abscess (%)	17 (1.4)	7 (1.6)	
Stasis (%)	18 (1.4)	11 (2.5)	
Pancreatic leakage (%)	14 (1.1)	0 (0.0)	
Ileus (%)	4 (0.3)	6 (1.4)	
Lymphorrhea (%)	14 (1.1)	3 (0.7)	
Wound infection (%)	2 (0.2)	5 (1.1)	
Others (%)	20 (1.6)	8 (1.8)	

### Subgroup analysis

Table 4 lists the clinical characteristics and pathologic features of the overall, non-obese, and obese patients whom underwent LAG and TLG. More patients in the LAG cohort underwent total gastrectomy. Besides, the TLG group had a more advanced tumor stage (*P* = 0.03). Otherwise, the two cohorts were similar with respect to age, gender, ASA score, and tumor size.

**Table 4:** Comparison of the clinicopathological characteristics in subgroup analysis

Variable	Overall			Non-obese			Obese		
	LAG (*n* = 724)	TLG (*n* = 967)	*P* value	LAG (*n* = 557)	TLG (*n* = 698)	*P* value	LAG (*n* = 167)	TLG (*n* = 269)	*P* value
Age (years)	58.7 ± 11.0	57.7 ± 11.0	0.08	58.6 ± 10.9	57.9 ± 11.2	0.26	59.1 ± 11.3	57.4 ± 10.7	0.12
Gender (M/F)	478/246	619/348	0.39	351/206	426/272	0.47	127/40	193/76	0.32
ASA(I/II/III)	339/353/32	480/439/48	0.39	259/276/22	334/334/30	0.82	80/77/10	146/105/18	0.36
Tumor size (cm)	3.7 ± 1.7	3.6 ± 1.7	0.22	3.7 ± 1.7	3.6 ± 1.6	0.36	3.8 ± 1.6	3.6 ± 1.7	0.40
TNM stage (I/II/III)	398/149/177	468/229/270	0.03	309/113/135	346/162/190	0.12	89/36/42	122/67/80	0.28
Surgical extension (D/T)	416/308	789/178	<0.01	308/249	561/137	<0.01	108/59	228/41	<0.01

*M* male, *F* female, *D* distal gastrectomy, *T* total gatrectomy

Surgical outcomes and postoperative recovery are shown in Table 5. Overall the mean operation time and blood loss were superior in the TLG group than those in the LAG group with significant differences (operation time: 220.2 ± 33.5 vs. 210.0 ± 25.6 min, *P* < 0.01, blood loss: 122.5 ± 39.8 vs. 117.1 ± 33.30 mL, *P* < 0.01). The numbers of retrieved lymph nodes were not significantly different between the two groups (34.7 ± 9.7 vs. 35.4 ± 9.4, *P* = 0.11). The time to first flatus and duration of stay were significantly lower in the TLG group (first flatus: 3.4 ± 0.9 vs. 3.3 ± 0.8 days, *P* = 0.02, hospital stay: 9.3 ± 2.6 vs. 9.0 ± 1.9 mL, *P* = 0.03). The time to starting food intake was also lower in the TLG group with a marginal difference (4.4 ± 0.9 vs. 4.3 ± 1.1 days, *P* = 0.08).

**Table 5:** Comparison of surgical outcomes and postoperative recovery in subgroup analysis

Variable	Overall			Non-obese			Obese		
	LAG (*n* = 724)	TLG (*n* = 967)	*P* value	LAG (*n* = 557)	TLG (*n* = 698)	*P* value	LAG (*n* = 167)	TLG (*n* = 269)	*P* value
Operation time (min)	220.2 ± 33.5	210.0 ± 25.6	<0.01	215.4 ± 32.9	205.4 ± 26.1	<0.01	236.0 ± 30.9	221.7 ± 20.0	<0.01
Blood loss (mL)	122.5 ± 39.8	117.1 ± 33.3	<0.01	115.0 ± 35.5	112.1 ± 33.0	0.14	147.5 ± 43.3	130.3 ± 30.2	<0.01
Number of RLN	34.7 ± 9.7	35.4 ± 9.4	0.11	34.6 ± 9.8	35.8 ± 9.8	0.04	34.9 ± 9.4	34.5 ± 8.3	0.64
Time to first flatus (d)	3.4 ± 0.9	3.3 ± 0.8	0.02	3.3 ± 0.8	3.2 ± 0.8	0.17	3.8 ± 0.9	3.5 ± 0.9	<0.01
Time to starting diet (d)	4.4 ± 0.9	4.3 ± 1.1	0.08	4.3 ± 0.9	4.3 ± 1.1	0.63	4.8 ± 1.0	4.5 ± 1.1	<0.01
Postoperative hospital stay (d)	9.3 ± 2.6	9.0 ± 1.9	0.03	9.1 ± 2.5	8.9 ± 2.0	0.12	9.9 ± 2.8	9.4 ± 1.8	0.03

*LAG* laparoscopic-assisted gastrectomy, *TLG* totally laparoscopic gastrectomy, *RLN* Regional lymph nodes

In the analysis of the obese subgroup, the operation time, blood loss, time to first flatus and diet, and duration of stay were also superior in the TLG group than in the LAG group (*P* < 0.05). However, in the non-obese subgroup analysis, only the operation time was significantly shorter in the TLG group, other parameters were not significantly different.

Table 6 presents postoperative complications in two groups. For all patients, the postoperative complication rate was somewhat lower in the TLG group, but the differences were not significant (10.6% vs. 8.9%, *P* = 0.23) and for non-obese patients, the postoperative complications were also not significantly different. However, in obese patients, the overall rate of complications was higher in the LAG cohort than in the TLG cohort with a significant difference (12.6% vs. 8.6%, *P* < 0.01). Especially, higher rates for ileus, stasis, and wound infection were observed in the LAG group.

**Table 6:** Comparison of postoperative complications in subgroup analysis

Variable	Overall			Non-obese			Obese		
	LAG (*n* = 724)	TLG (*n* = 967)	*P* value	LAG (*n* = 557)	TLG (*n* = 698)	*P* value	LAG (*n* = 167)	TLG (*n* = 269)	*P* value
Overall complications (%)	77 (10.6)	86 (8.9)	0.23	56 (10.1)	63 (9.0)	0.54	21 (12.6)	23 (8.6)	<0.01
Anastomotic leakage (%)	2 (0.3)	4 (0.4)		2 (0.4)	3 (0.4)		0 (0.0)	1 (0.4)	
Anastomotic stricture (%)	3 (0.4)	3 (0.3)		3 (0.5)	3 (0.4)		0 (0.0)	0 (0.0)	
Anastomotic bleeding (%)	4 (0.6)	5 (0.5)		4 (0.7)	4 (0.6)		0 (0.0)	1 (0.4)	
Intracorporeal hemorrhage (%)	7 (1.0)	6 (0.6)		6 (1.1)	5 (0.7)		1 (0.6)	1 (0.4)	
Abdominal abscess (%)	9 (1.2)	15 (1.6)		6 (1.1)	11 (1.6)		3 (1.8)	4 (1.5)	
Stasis (%)	12 (1.7)	17 (1.8)		7 (1.3)	11 (1.6)		5 (3.0)	6 (2.2)	
Pancreatic leakage (%)	7 (1.0)	7 (0.7)		7 (1.3)	7 (1.0)		0 (0.0)	0 (0.0)	
Ileus (%)	6 (0.8)	4 (0.4)		2 (0.4)	2 (0.3)		4 (2.4)	2 (0.7)	
Lymphorrhea (%)	9 (1.2)	8 (0.8)		7 (1.3)	7 (1.0)		2 (1.2)	1 (0.4)	
Wound infection (%)	5 (0.7)	2 (0.2)		2 (0.4)	0 (0.0)		3 (1.8)	2 (0.7)	
Others (%)	13 (1.8)	15 (1.6)		10 (1.8)	10 (1.4)		3 (1.8)	5 (1.9)	

*LAG* laparoscopic-assisted gastrectomy, *TLG* totally laparoscopic gastrectomy

Tables 7 and 8 present the surgical outcomes and postoperative recovery for distal or total gastrectomy, respectively. Similar to Table 6, in obese group, regardless of LAG subgroup or TLG subgroup, the surgical and perioperative outcomes were favorable in TLG. But in the non-obese group, these advantages were not obvious.

**Table 7:** Comparison of surgical outcomes, postoperative recovery and postoperative complications in Subgroup analysis for distal gastrectomy

Variable	Non-obese			Obese		
	LADG(*n* = 308)	TLDG(*n* = 561)	*P* value	LADG(*n* = 108)	TLDG(*n* = 228)	*P* value
Operation time (min)	198.5 ± 21.1	198.7 ± 21.0	0.89	227.6 ± 25.2	218.2 ± 17.7	<0.01
Blood loss (mL)	107.2 ± 26.6	110.0 ± 32.8	0.17	141.1 ± 44.8	129.0 ± 30.7	<0.01
Number of RLN	34.7 ± 8.5	35.5 ± 9.7	0.20	33.9 ± 9.7	34.1 ± 8.7	0.88
Time to first flatus (d)	3.2 ± 0.8	3.2 ± 0.8	0.90	3.8 ± 1.0	3.5 ± 0.9	0.02
Time to starting diet (d)	4.2 ± 0.9	4.2 ± 0.9	0.67	4.8 ± 1.0	4.5 ± 1.0	0.01
Postoperative hospital stay (d)	8.7 ± 2.0	8.8 ± 1.9	0.81	9.8 ± 2.6	9.4 ± 1.8	0.13
Overall complications (%)	26 (8.4)	44 (7.8)	0.80	13 (12.0)	18 (7.9)	0.22
Anastomotic leakage (%)	1 (0.3)	2 (0.4)		0 (0.0)	1 (0.4)	
Intracorporeal hemorrhage (%)	3 (1.0)	4 (0.7)		1 (0.9)	1 (0.4)	
Anastomotic bleeding (%)	1 (0.3)	2 (0.4)		0 (0.0)	1 (0.4)	
Abdominal abscess (%)	3 (1.0)	8 (1.4)		2 (1.9)	3 (1.3)	
Stasis (%)	5 (1.6)	9 (1.6)		3 (2.8)	5 (2.2)	
Pancreatic leakage (%)	4 (1.3)	5 (0.9)		0 (0.0)	0 (0.0)	
Ileus (%)	0 (0.0)	1 (0.2)		3 (2.8)	1 (0.4)	
Lymphorrhea (%)	3 (1.0)	6 (1.1)		1 (0.9)	1 (0.4)	
Wound infection (%)	1 (0.3)	0 (0.0)		1 (0.9)	1 (0.4)	
Others (%)	5 (1.6)	7 (1.2)		2 (1.9)	4 (1.8)	

*LADG* laparoscopic-assisted distal gastrectomy, *TLDG* totally laparoscopic distal gastrectomy, *RLN* Regional lymph nodes

**Table 8:** Comparison of surgical outcomes, postoperative recovery and postoperative complications in Subgroup analysis for total gastrectomy

Variable	Non-obese			Obese		
	LATG(*n* = 249)	TLTG(*n* = 137)	*P* value	LATG(*n* = 59)	TLTG(*n* = 41)	*P* value
Operation time (min)	236.4 ± 32.8	233.1 ± 26.8	0.32	251.4 ± 34.5	241.5 ± 20.7	0.10
Blood loss (mL)	124.7 ± 42.1	120.5 ± 32.7	0.28	159.2 ± 38.2	137.6 ± 26.7	<0.01
Number of RLN	34.5 ± 11.1	36.8 ± 10.1	0.04	36.7 ± 8.5	36.8 ± 5.2	0.96
Time to first flatus (d)	3.4 ± 0.8	3.3 ± 0.7	0.21	3.9 ± 0.8	3.5 ± 0.9	0.04
Time to starting diet (d)	4.4 ± 0.9	4.4 ± 1.5	0.96	4.9 ± 0.8	4.6 ± 1.2	0.15
Postoperative hospital stay (d)	9.5 ± 2.9	9.3 ± 2.1	0.55	10.1 ± 3.0	9.3 ± 1.6	0.15
Overall complications (%)	30 (12.0)	19 (13.9)	0.61	8 (13.6)	5 (12.2)	0.84
Anastomotic leakage (%)	1 (0.4)	1 (0.7)		0 (0.0)	0 (0.0)	
Anastomotic stricture (%)	3 (1.2)	3 (2.2)		0 (0.0)	0(0.0)	
Anastomotic bleeding (%)	3 (1.2)	2 (1.5)		0 (0.0)	0 (0.0)	
Intracorporeal hemorrhage (%)	3 (1.2)	1 (0.7)		0 (0.0)	0 (0.0)	
Abdominal abscess (%)	3 (1.2)	3 (2.2)		1 (1.7)	1 (2.4)	
Stasis (%)	2 (0.8)	2 (1.5)		2 (3.4)	1 (2.4)	
Pancreatic leakage (%)	3 (1.2)	2 (1.5)		0 (0.0)	0 (0.0)	
Ileus (%)	2 (0.8)	1 (0.7)		1 (1.7)	1 (2.4)	
Lymphorrhea (%)	4 (1.6)	1 (0.7)		1 (1.7)	0 (0.0)	
Wound infection (%)	1 (0.4)	0 (0.0)		2 (3.4)	1 (2.4)	
Others (%)	5 (2.0)	3 (2.2)		1 (1.7)	1 (2.4)	

*LATG* laparoscopic-assisted total gastrectomy, *TLTG* totally laparoscopic total gastrectomy, *RLN* Regional lymph nodes

## Discussion

Despite a decrease in incidence, gastric adenocarcinoma is still the third most deadly cancer worldwide and surgical resection and proper perigastric lymphadenectomy is the only treatment option for increasing the survival rate [1, 40]. Laparoscopic surgery has been proposed as a promising method for the therapy of gastric cancer [41, 42]. Another fact is that people around the world are getting fatter. Furthermore, obesity is no longer just found in wealthy nations. It is now a worldwide problem and the rate has almost doubled since the 1980s [19]. Therefore, there is an increasing need to improve minimally invasive approaches for obese gastric cancer patients.

Our results suggest that LG in obese or overweight gastric cancer patients poses an increased technical challenge as demonstrated by longer operating time, increased blood loss, and later recovery compared to non-obese patients. Clearly, because of the hindered exposure to the pancreas and stomach, surgery in obese patients is more demanding technically. Under particular circumstances, the thickened omentum, mesentery and ligamentum may result in challenges in ligation, mobilization, or dissection of the lymph nodes and vessels. The fatty omentum and stomach itself can cause serious difficulties when the stomach is pulled by the assistant, excessive fat and incrassate mesenteries can also lead to problematic hemorrhage, which is difficult to stop in the narrow area caused by the adipose tissue. Presumably, due to visceral fat in obese patients, the enhanced technical difficulties and the exposure of an adequate operative field play a role in the distribution of anaesthetic agents which may lead to delayed sensation recovery from anaesthesia. All of the above inevitably increase surgical duration and blood loss. Also, the longer incision needed due to increased abdominal wall thickness in obese patients results in more postoperative pain, which inevitably lead to higher frequency of painkiller usage [43]. Subsequent gastrointestinal recovery, delayed postoperative activities, prolonged time of abdominal cavity exposure, and increased usage of analgesic drugs were the major reasons for extended postoperative hospital stay duration.

The rate of morbidity generally grew amongst obese patients; however, such difference was insignificant (9.5% vs. 10.1%). The relatively higher rate of overall complications in the obese cohort was due mainly to a greater incidence of wound infection, ileus and stasis. In these patients, the higher probability of wound infection may be associated with local factors, like the deeper abdominal wall and the need for longer auxiliary incisions. Nonetheless, systemic factors, for instance, poor glycaemic control and greater insulin resistance, may be involved, which can lead to greater susceptibility to bacteria. The increased ileus and stasis might be partly due to the technically difficult manipulating the fatty stomach and bowels for resection and reconstruction. After surgery, another explanation for the association of obesity with stasis and ileus is a delay or decrease in ambulation [44].

Our findings are concordant with the results of other large studies from Asian countries regardless of open or laparoscopic abdominal surgery [45, 46]. However, some reports had inconsistent results mainly with Western countries. According to some American studies, overweight and mildly obese patients even tended to have better outcomes than the normal weight patients [47, 48]. Such difference could be ascribed to insufficient sample size to identify statistically significant differences. Another contributor to the discrepancy may be the inexperience of surgeons in East Asia in treating obese patients in comparison with their Western counterparts, because, in general, the average BMI for Asian people is much lower than that of Western people, despite its recent increase.

Based on our findings, LG, though less invasive compared to conventional open surgery, is still more traumatic for obese gastric cancer patients than non-obese patients. Therefore, a relatively less invasive approach for obese gastric cancer patients is urgently needed. Lee et al. reported that the advantages of robotic gastrectomy were less optimal with normal weight patients characterized by less blood loss than in over-weight patients [49]. Therefore, he argued that patients with high BMI may be good candidates for robotic surgery when deciding between minimally invasive approaches for curing gastric cancer. However, compared to operations such as rectal or prostatic surgery, which are in relatively narrow regions, gastric surgery located on the upper abdomen is relatively spacious, the superiority of the da Vinci robotic proceedure over laparoscopy is not obvious [50–52]. In addition, the downside of added costs and longer operation times compared with a laparoscopic approach is also an important consideration [53]. Because the heath care resources for Chinese patients are limited, they need to self-pay for such a costly system, it is more sensible to enhance the laparoscopic approach for obese patients rather than using the da Vinci robot.

It is well-known that there are two methods of LG with the main differences that anastomoses are performed intracorporeally or extracorporeally. TLG is regarded as incisionless, with only minimal trocar wounds and characterized by such operation of in situ, no tumor touch, more selectivity and dexterity for surgeons and total direct vision during operation [37]. These advantages may help get over the limitations of laparoscopic surgery in obese patients.

Though some observational studies and meta-analysis reported favorable outcomes of TLG compared to LAG [54–59], there are still some studies, which included randomized controlled trials (RCTs), that failed to identify clinical advantages of TLG over LAG [60–66], however these studies did not evaluate the benefit or superiority of TLG over LAG in obese patients. Also, based on our data of non-obese patients, the outcomes of TLG are not inferior to those of LAG, but for obese patients, TLG has several advantages over LAG, such as shortened operating time, less intraoperative bleeding, earlier recovery, and fewer complications. After further analysis by dividing TLG into TLDG and TLTG, findings were similar. Our results mean that TLG could be more favorable than LAG among obese patients regardless of total or distal gastrectomy. However, given the small number of patients within this subgroup, especially in total gastrectomy of obese patents, these results should be interpreted with caution. Further studies are warranted before drawing definitive conclusions.

There are several limitations in our study. The selection bias is the most significant limitation of this research. There were great differences between obese and non-obese cohorts in patient comorbidities and surgical extensions. During earlier trials when we performed LG or at the beginning of a junior carrying out LG, surgeons often chose LAG, then started to attempt TLG only after a sufficient accumulation of laparoscopic experience. This could have affected our results. The retrospective nature at a single academic institution is another limitation. Thus, patient heterogeneity might be a potential confounder in this research. Due to the differences in operative indications and populations, direct comparisons of non-obese and obese patients seem inappropriate methodologically, when retrospective data are used. Possible sources of heterogeneity included surgeon experience, variable medical group habits and small sample sizes. Also this study only examined the short-term outcomes following LG for gastric cancer. The long-term effects of overweight and obesity on cancer mortality and quality of life remain unknown.

## Conclusions

In conclusion, our study suggests that LG for gastric cancer in the obese poses an increased technical challenge as demonstrated by longer operating time, increased blood loss and later postoperative recovery compared to non-obese patients. TLG may offer several advantages compared to LAG including operation of in situ, no tumor touch, improved visualization and dexterity, which may improve outcomes, thus it is more suitable for obese patients. Future studies should focus on collecting robust prospective data that compares short-term surgical and long-term survival results in the obese for each of the existing minimally invasive operation options.

## Abbreviations

ASA: American Society of Anesthesiologists
BMI: Body mass index
EBL: Estimated blood loss
LADG: Laparoscopic-assisted distal gastrectomy
LAG: Laparoscopic-assisted gastrectomy
LATG: Laparoscopic-assisted total gastrectomy
LG: Laparoscopic gastrectomy
RCT: Randomized controlled trial
SD: Standard deviations
TLDG: Totally laparoscopic distal gastrectomy
TLG: Totally laparoscopic gastrectomy
TLTG: Totally laparoscopic total gastrectomy
TNM: Tumor-node-metastasis
